# High-Capacitance Gold Nanoparticles from *Rhus coriaria*: Green Synthesis, Characterization and Electrochemical Evaluation for Supercapacitor Technologies

**DOI:** 10.3390/mi17010082

**Published:** 2026-01-08

**Authors:** Mehmet Firat Baran, Elchin Huseynov, Aziz Eftekhari, Abdulkadir Levent, Erdal Ertaş, Taras Kavetskyy, Ondrej Šauša, Evgeny Katz, Oleh Smutok

**Affiliations:** 1 Department of Food Technology, Vocational School of Technical Sciences, Batman University, 72000 Batman, Turkey; erdalertas21@gmail.com; 2 Department of Infectious Diseases, Azerbaijan Medical University, AZ1022 Baku, Azerbaijan; huseynovel@amu.az; 3 Department of Biochemistry, Faculty of Science, Ege University, 35040 Izmir, Turkey; aziz.eftekharimehrabad@ege.edu.tr; 4 Department of Chemistry, Faculty of Arts and Sciences, Batman University, 72100 Batman, Turkey; levent.kadir@batman.edu.tr; 5 Department of Physics and Information Systems, Drohobych Ivan Franko State Pedagogical University, 82100 Drohobych, Ukraine; kavetskyy@yahoo.com; 6 Department of Biology and Chemistry, Drohobych Ivan Franko State Pedagogical University, 82100 Drohobych, Ukraine; 7 Institute of Physics, Slovak Academy of Sciences, 84511 Bratislava, Slovakia; ondrej.sausa@savba.sk; 8 Department of Nuclear Chemistry, Faculty of Natural Sciences, Comenius University, 84215 Bratislava, Slovakia; 9 Department of Chemistry and Biochemistry, Clarkson University, Potsdam, NY 13699, USA; ekatz@clarkson.edu

**Keywords:** renewable energy technologies, *Rhus coriaria*, Rc@AuNPs, energy storage, electrolyte, capacitive behavior

## Abstract

The structural and electrochemical properties of gold nanoparticles biosynthesized from *Rhus coriaria* L. (Rc@AuNPs) were comprehensively investigated and characterized. *R. coriaria* (sumac) served as a natural gold reducing and capping agent due to its rich polyphenolic and phytochemical composition, enabling the sustainable, low-cost, and environmentally friendly synthesis of Rc@AuNPs. The electrochemical behavior of the hybrid material was evaluated using cyclic voltammetry (CV), galvanostatic charge–discharge (GCD), and electrochemical impedance spectroscopy (EIS). Rc@AuNPs exhibited specific capacitances of 129.48 F/g, 156.32 F/g, and 280.37 F/g in H_2_SO_4_, Na_2_SO_4_, and KOH electrolytes, respectively, indicating strong potential for supercapacitor and energy-storage applications. GCD analysis further showed *C_sp_* values of 107.69 F/g (H_2_SO_4_), 133.23 F/g (Na_2_SO_4_), and 348.34 F/g (KOH), confirming the highest charge-storage performance in basic media. EIS measurements supported these results, yielding equivalent series resistance (ESR) values of 67.96 Ω in H_2_SO_4_, 64.42 Ω in Na_2_SO_4_, and a notably lower 24.43 Ω in KOH, consistent with its higher ionic conductivity and more efficient charge transfer. Overall, the superior *C_sp_* and low ESR observed in KOH demonstrate the excellent capacitive behavior of Rc@AuNPs. These biosynthesized gold nanoparticles represent a promising and sustainable electrode material for high-performance energy-storage technologies.

## 1. Introduction

Global temperature rise and climate instability are direct consequences of human-driven greenhouse gas emissions. The intensive consumption of fossil fuels not only threatens energy security but also causes irreversible ecological damage. The increasing atmospheric concentrations of CO_2_, CH_4_, and N_2_O accelerate global warming, leading to sea-level rise, extreme weather events, biodiversity loss, and declining agricultural productivity. These challenges underscore the limitations of fossil fuel-based energy systems and highlight the urgent need for renewable and sustainable energy technologies [1,2,3]. As renewable energy sources such as solar, wind, biomass, and hydrogen become more widely deployed, the development of advanced energy-storage systems, including batteries and supercapacitors, has become essential for safe and efficient energy utilization. Consequently, environmentally friendly, cost-effective, and high-performance energy technologies have emerged as a scientific and technological priority to enhance energy security, reduce carbon emissions, and achieve global sustainability targets [4,5,6].

Supercapacitors are particularly attractive due to their high-power density, rapid charge–discharge rates, long cycle life, and excellent electrochemical stability [7,8]. They are classified into three main categories based on their charge-storage mechanisms: electrical double-layer capacitors (EDLCs), pseudocapacitors, and hybrid capacitors [9,10]. EDLCs store energy through electrostatic charge separation at the electrode–electrolyte interface, whereas pseudocapacitors rely on fast, reversible surface or bulk redox reactions [11,12,13]. Hybrid supercapacitors integrate both mechanisms to achieve higher energy and power densities. In all cases, optimizing the morphological, chemical, and conductive properties of electrode materials is a key determinant of overall performance [14,15,16].

In recent years, metal nanoparticles produced via green synthesis have gained attention for energy-storage and biosensing applications due to their sustainability, low cost, and environmental compatibility. These methods employ plant extracts as natural reducing and capping agents, eliminating toxic reagents and enabling the fabrication of biocompatible nanomaterials [17,18]. *Rhus coriaria* L. (sumac) is particularly notable for its high content of phenolics, flavonoids, and tannins, which effectively reduce metal ions and stabilize nanoparticle formation. Gold nanoparticles synthesized using *R. coriaria* extract (Rc@AuNPs) exhibit high electrical conductivity and chemical stability, making them promising candidates for both sustainable energy-storage applications and biosensors [19,20].

Gold nanoparticles are widely utilized due to their strong surface plasmon resonance (SPR), high conductivity, and excellent affinity toward biomolecules, which enable highly sensitive biosensing. Their large electrochemically active surface area also promotes efficient ion transport, contributing to improved specific capacitance in supercapacitor systems [21,22,23]. Thus, Rc@AuNPs produced via green synthesis represent multifunctional nanomaterials with strong potential in both high-performance energy storage and biosensing. This environmentally friendly approach advances the integration of bio-derived nanomaterials into future sustainable energy and biomedical technologies.

## 2. Materials and Methods

### 2.1. Preparation of Rhus coriaria L. Plant Extract

Seeds of *Rhus coriaria* L., commercially obtained from the Hasankeyf district of Batman, were washed several times with tap water and subsequently rinsed with distilled water. After drying at room temperature, the seeds were ground into a fine powder. A total of 40 g of this powder was mixed with 600 mL of distilled water and boiled. The resulting extract was cooled to room temperature and filtered through Whatman No. 1 filter paper.

### 2.2. Gold Nanoparticles (Rc@AuNPs) Synthesis

For nanoparticle synthesis, a 100 mg/L solution of Sigma-Aldrich tetrachloroauric acid (HAuCl_4_·3H_2_O) was prepared. Then, 200 mL of the *R. coriaria* extract was combined with the prepared 100 ppm gold solution. The reaction mixture was stirred at 60 °C for 2 h. Following the reaction, the dark-colored solution was centrifuged, and the obtained solid was dried in an oven at 110 °C for 48 h. The resulting gold nanomaterials (Rc@AuNPs) were collected and stored for subsequent electrochemical analyses.

### 2.3. Electrodes Preparation

The gold nanoparticle-coated electrode material (Rc@AuNPs) was prepared using a sustainable green synthesis method, taking advantage of the reducing properties of bioactive compounds in *R. coriaria* plant extract. The resulting Rc@AuNP nanocomposite was used as the active component of the electrode. A measured amount of graphite powder was added to increase the electrical conductivity of the composite, and mineral oil was used as the binder phase. The mixture for the electrode material was blended until a homogeneous paste was obtained, using a ratio of 75% active component (Rc@AuNPs), 5% graphite, and 20% binder (7.5:0.5:2, *w*/*w*) [24]. Based on this composition, approximately 10 mg of Rc@AuNPs was loaded onto each electrode.

The prepared nanocomposite paste was carefully packed into the cavity of a commercial carbon paste electrode (CPE; MF-2010, BASi, Edinburgh, UK), and its surface was smoothed and compacted to make it suitable for electrochemical analysis.

### 2.4. Electrochemical Measurements

All electrochemical measurements were carried out using an AUTOLAB PGSTAT128N potentiostat/galvanostat system. A three-electrode cell configuration manufactured by BASi was used for the experiments. In this electrochemical setup, a platinum wire (Pt; MF-1032, BASi) was used as the auxiliary electrode, and an Ag/AgCl (3 M KCl) electrode (MF-1063, BASi) served as the reference electrode.

The energy storage properties of the designed supercapacitor were investigated in various supporting electrolyte environments (1 M H_2_SO_4_, Na_2_SO_4_, and NaOH). The electrochemical properties of the capacitor were comprehensively analyzed using cyclic voltammetry (CV), galvanostatic charge–discharge (GCD), and electrochemical impedance spectroscopy (EIS) methods.

### 2.5. Calculation of Specific Capacitance

The energy storage performance of the Rc@ZnONPs electrode was assessed through calculation of the specific capacitance (*C_sp_*) using three complementary electrochemical techniques: cyclic voltammetry (CV), galvanostatic charge–discharge (GCD), and electrochemical impedance spectroscopy (EIS). Measurements were conducted using either a three-electrode system or a symmetric two-electrode configuration, depending on the experimental setup. The corresponding calculations were performed based on well-established equations commonly reported in the literature [25].

(1) From CV measurements: $C_{sp}=\frac{A}{2r∆Vm}$
(2) From GCD curves: $C_{sp}=\frac{It_{d}}{∆Vm}\;$ (for three-electrode)
(3) From GCD curves: $C_{sp}=\frac{It_{d}}{2∆Vm}\;$ (for two-electrode)
(4) From EIS data: $\;C_{sp}=\frac{-1}{2\pi fmZ^{\prime \prime }}$

where *A* is the integrated area under the CV curve (A·V), *r* is the potential scan rate (V/s), Δ*V* is the potential window (V), *I* is the discharge current (A), *t_c_* and *t_d_* are the charge and discharge times (s), respectively, m represents the mass of the active electrode material (g), ƒ is the frequency (Hz), and *Z*″ is the imaginary component of impedance (Ω).

In addition to the specific capacitance, the energy density (E_s_, Wh/kg) and power density (P_s_, W/kg) of the electrode were calculated as follows:

(5)
$E_{s}\;(Wh/kg)=\frac{1}{2}\frac{Csp{\left(∆V\right)}^{2}}{3.6}$

(6)
$P_{s}(Wh/kg)=\frac{Ed}{t_{d}}3600$

## 3. Results and Discussion

### 3.1. Characterization of Rc@AuNPs

#### 3.1.1. UV-Vis Analysis

The color of the reaction solution changed from light yellow to ruby red upon the synthesis of Rc@AuNPs using *R. coriaria* L. extract, indicating the formation of gold nanoparticles. This color transition is attributed to the surface plasmon (SP) vibrations of Rc@AuNPs. The optical properties of metal nanoparticles are strongly dependent on their size and shape. According to Mie theory, small spherical gold nanoparticles exhibit a single surface plasmon resonance (SPR) absorption band, whereas anisotropic gold nanoparticles may display two or three SPR bands [26].

**Figure 1:**
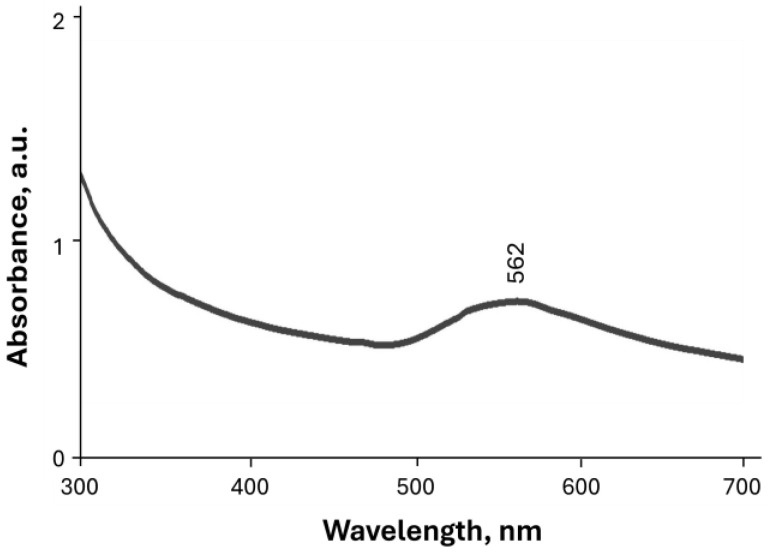
UV-vis spectrum of Rc@AuNP nanoparticles.

The UV–Vis spectrum of Rc@AuNPs is shown in Figure 1. A distinct SPR absorption peak centered at 562 nm was observed, confirming the successful formation of gold nanoparticles [27].

#### 3.1.2. FTIR Analysis

**Figure 2:**
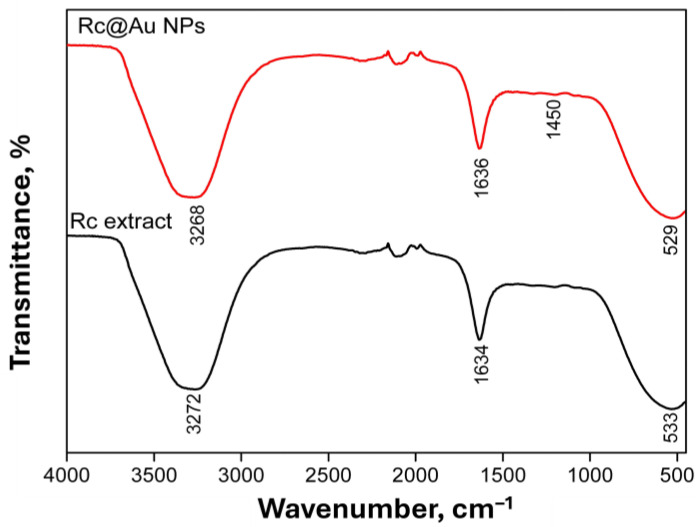
FTIR spectra of *R. coriaria* extract and Rc@AuNP nanoparticles.

Phytochemicals such as flavonoids, tannins, alkaloids, terpenoids, and polyphenols present in the *R. coriaria* plant extract act as reducing and stabilizing agents during the synthesis of Rc@AuNPs. The FTIR spectra of the *R. coriaria* extract and Rc@AuNPs are shown in Figure 2. Characteristic absorption bands observed at 1634 and 1636 cm^−1^ correspond to the C=O stretching vibration of carbonyl groups associated with carboxylic acids in the *R. coriaria* extract and Rc@AuNPs, respectively.

For Rc@AuNPs, the O–H bending vibration associated with carboxylic acid groups was observed at 1450 cm^−1^. In addition, broad absorption bands corresponding to the O–H stretching vibrations of hydroxyl groups were detected at 3372 and 3368 cm^−1^ for the *R. coriaria* extract and Rc@AuNPs, respectively (Figure 2), indicating the involvement of hydroxyl-containing phytochemicals in the reduction and stabilization of the gold nanoparticles [28].

#### 3.1.3. SEM Analysis

**Figure 3:**
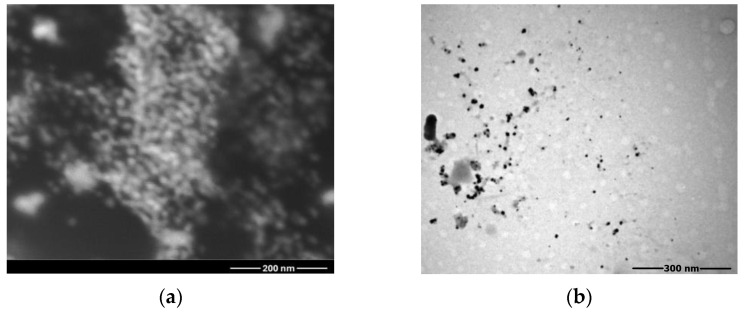
(**a**) SEM and (**b**) TEM images of Rc@AuN nanoparticles.

The surface morphology of Rc@AuNPs was examined using SEM and TEM at different magnifications. As shown in the SEM (Figure 3a) and TEM (Figure 3b) images, the nanoparticles exhibit diverse morphologies, including spherical, triangular, hexagonal rod-like, and cubic shapes. The presence of these varied geometries indicates the polydisperse nature of the synthesized Rc@AuNPs.

The relatively high concentration of phytocomponents in the *R. coriaria* extract may have promoted partial aggregation during nanoparticle formation, contributing to the predominantly spherical morphology of Rc@AuNPs [29].

#### 3.1.4. EDX Analysis

**Figure 4:**
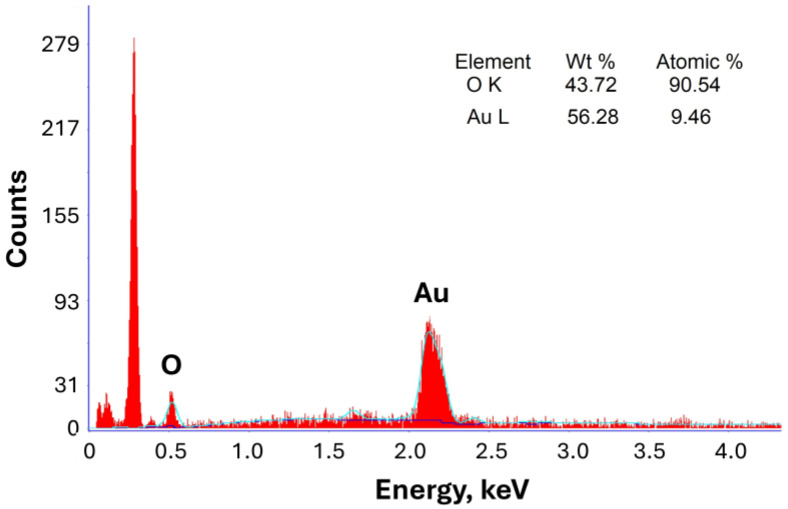
EDX spectrum of Rc@AuNP nanoparticles.

EDX analysis confirmed the successful synthesis and elemental composition of Rc@AuNPs produced via green synthesis using *R. coriaria* L. extract, as shown in Figure 4.

A strong and characteristic Au signal was observed at approximately 2.25 keV, indicating the presence of gold nanoparticles. In addition, weak peaks corresponding to oxygen were detected, which can be attributed to phytochemical compounds adsorbed on the surface of the Au nanoparticles, originating from the *R. coriaria* extract [28].

#### 3.1.5. XRD Analysis

**Figure 5:**
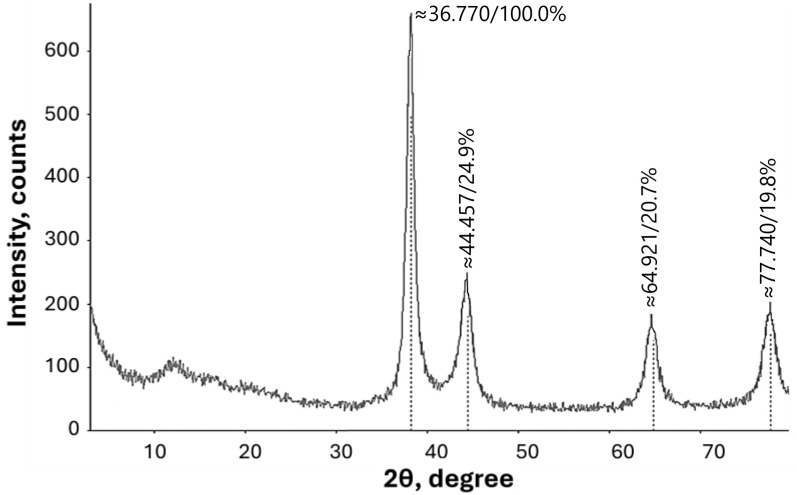
X-ray diffractograms of Rc@AuNP nanoparticles.

The crystalline structure of Rc@AuNP nanoparticles was analyzed using X-ray diffraction (XRD), as shown in Figure 5. The average crystallite size of the Au nanoparticles was calculated using the Debye–Scherrer equation and found to be approximately 19.40 nm [30].

The XRD pattern exhibited distinct Bragg reflection peaks at 2θ values of 38°, 44°, 64°, and 77°, corresponding to the (111), (200), (220), and (311) crystal planes, respectively. These peaks closely match the standard XRD data for gold (JCPDS Card No. 00–001-1172), confirming the formation of crystalline gold nanoparticles. The results indicate that the green-synthesized Rc@AuNPs possess a face-centered cubic (fcc) structure, consistent with the typical crystallinity of gold [31].

#### 3.1.6. Zeta Potential Analysis

**Figure 6:**
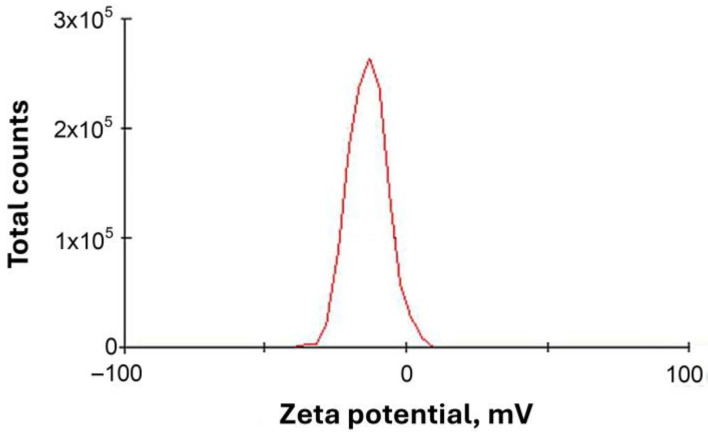
Zeta potential of Rc@AuNP nanoparticles, indicating their surface charge and colloidal stability.

The colloidal Rc@AuNP nanoparticles synthesized using *R. coriaria* extract exhibited a zeta potential of −13.5 mV (Figure 6).

This negative surface charge is attributed to the adsorption of phytochemicals from the plant extract onto the nanoparticle surface. These phytocomponents act as both reducing and stabilizing agents, generating electrostatic repulsion between nanoparticles that helps prevent aggregation [31].

### 3.2. Cyclic Voltammetry of the Rc@AuNPs-Modified Electrode

**Figure 7:**
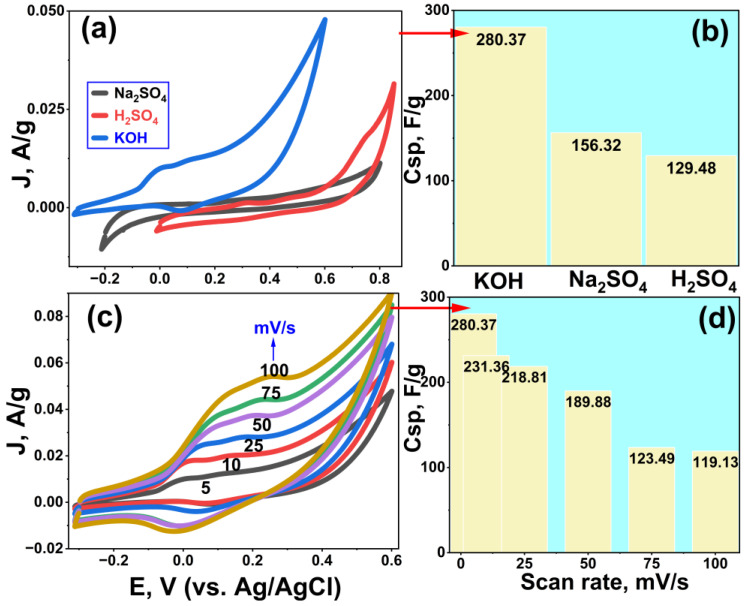
Electrochemical performance of Rc@AuNPs: (**a**) CV curves in H_2_SO_4_, Na_2_SO_4_, and KOH (5 mV/s), (**b**) corresponding specific capacitances, (**c**) CV curves in KOH at varying scan rates, and (**d**) scan rate-dependent specific capacitances.

The electrochemical behavior of the Rc@AuNPs electrode material was investigated in different electrolyte solutions (H_2_SO_4_—red curve, Na_2_SO_4_—black curve, and KOH—blue curve) at a scan rate of 5 mV/s (Figure 7a). The overall profile of the CV curves exhibits a rectangular shape typical of supercapacitors, with the blue curve in KOH showing a larger current area. This suggests that the Rc@AuNPs electrode exhibits a more pronounced pseudocapacitive behavior in KOH solution. The high specific capacitance value of 280.37 F/g in KOH is attributed not only to electrochemical double-layer capacitance (EDLC) but also to the pseudocapacitance effect resulting from surface redox processes. Pseudocapacitance is characterized by reversible surface redox reactions or electrode/ion interactions (for example, the literature reports an increase in pseudocapacitance when Au NPs are applied to Ni(OH)_2_ electrodes) [32].

The basis for this contribution is the electroactive behavior of Au nanoparticles (AuNPs) in alkaline media. In alkaline solutions, a redox transformation can occur on the Au surface as follows:

Au + OH^−^ ⇋ Au–OH + e^−^

This process leads to the formation of Au–OH species on the surface, particularly in more positive potential regions. The resulting Au–OH and its derivatives facilitate rapid and reversible charge transfer across the electrode surface. Therefore, the high capacitance values observed in KOH can be interpreted as hybrid capacitance behavior, contributed by double-layer capacitance (EDLC) and surface redox reactions (Au/Au–OH transition).

In contrast, due to the lower concentration of OH^−^ ions and limited interaction with the Au surface in neutral (Na_2_SO_4_) and acidic (H_2_SO_4_) environments, AuNPs are less able to exhibit the same electroactive behavior. In this context, the influence of the electrolyte environment on AuNP–electrode interactions has been discussed in the literature [33,34]. This is consistent with the specific capacitance order KOH > Na_2_SO_4_ > H_2_SO_4_ shown in Figure 7b.

CV curves (Figure 7c), obtained at different scan rates (5–100 mV/s), provide additional information about ion transport and electrode kinetics. At lower scan rates, ions reach the active sites more easily, allowing both EDLC and pseudocapacitive contributions to participate [35]. However, due to limited ion diffusion time at high scan rates, pseudocapacitive reactions cannot fully occur, and therefore the C_sp_ value (Figure 7d) decreases from 280.37 F/g to 119.13 F/g. This decrease indicates that the Rc@AuNPs material exhibits hybrid-capacitor behavior controlled by ion diffusion-dependent redox processes. Furthermore, the literature supports that AuNP doping increases electrode conductivity and charge-transfer rate; for instance, AuNPs have been shown to increase conductivity in carbon/metal-oxide electrode systems [36]. This may promote pseudocapacitive activity by accelerating electrochemical kinetics.

Based on the data presented in Figure 7c, the electrochemical responses of the Rc@AuNPs-modified electrode at different scan rates were systematically analyzed. The relationships between the scan rate and the anodic peak current (*Ip*) as well as the anodic peak potential were quantitatively evaluated. A linear relationship was observed between the anodic peak current and the scan rate, described by the equation:

$$I_{p}\left(\mu A\right)=0.0012\;v\;\left(mV/s\right)+5.48\;\left(r=0.993\right)$$

Similarly, the relationship between the anodic peak current and the square root of the scan rate was expressed as:

$$I_{p}\left(\mu A\right)=4.84\;\sqrt{v\;(mV/s)}-18.142\;(r=0.996)$$

In addition, the logarithmic correlation between the anodic peak current and the scan rate was evaluated:

$$\log I_{p}\left(\mu A\right)=0.67\log v\;\left(mV/s\right)+0.61$$

The slope of ~0.65 obtained from this log–log plot indicates that the electrochemical process is governed by a mixed control mechanism, with contributions from both diffusion-controlled and adsorption-controlled processes. These results demonstrate that the Rc@AuNPs-modified electrode exhibits predominantly diffusion-controlled behavior in an alkaline medium.

Thus, the highly electroactive behavior of the Rc@AuNPs supercapacitor in KOH medium is associated with the efficient adsorption of OH^−^ ions on the Au surface and the rapid redox conversion of Au–OH species [30,37]. Moreover, Rc@AuNPs synthesized via a biological route are expected to possess a heterogeneous surface structure, comprising different crystallographic facets, surface defects, and grain boundaries. Each of these discrete surface regions exhibits distinct thermodynamic and kinetic properties, resulting in redox processes distributed over a wide potential range and the appearance of multiple redox signals. Additionally, biomass-derived organic components (e.g., polyphenols, flavonoids) from the *R. coriaria* extract, adsorbed on the Au nanoparticle surface, may exhibit limited electrochemical activity at various potentials. Given the available data, it is challenging to unambiguously assign each observed redox peak to a specific molecular or surface event. Instead, the multiple redox signals likely arise from the combined effects of Au/Au–OH surface transformations, heterogeneous surface characteristics, and biomass-derived functional groups. This behavior suggests that Rc@AuNPs electrodes possess a gradual and rich faradaic charge storage capability across a wide potential window, which is consistent with the high capacitance values obtained. This feature demonstrates that the material exhibits a pseudocapacitive supercapacitor behavior that goes beyond the classical double-layer storage mechanism and provides enhanced energy density and strong cycling stability.

### 3.3. Charge–Discharge Behavior of the Rc@AuNPs Electrode

**Figure 8:**
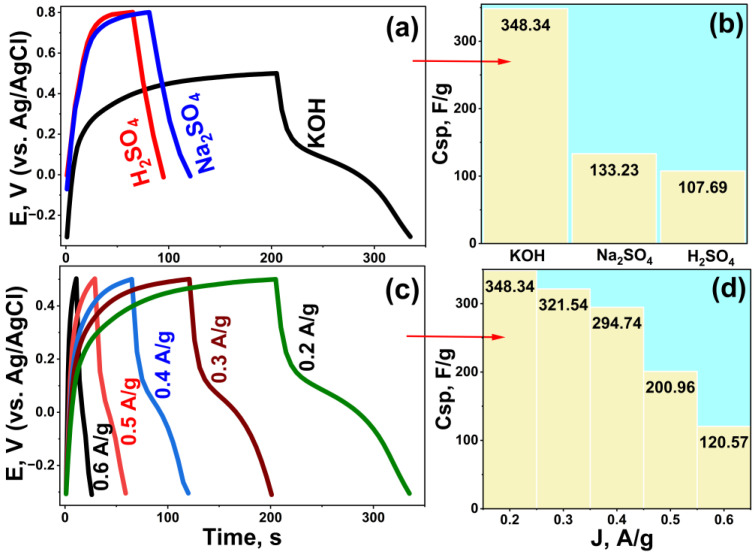
Electrochemical performance of Rc@AuNPs-based supercapacitor: (**a**) galvanostatic charge–discharge (GCD) curves in H_2_SO_4_, Na_2_SO_4_, and KOH electrolytes; (**b**) corresponding specific capacitance values in each electrolyte; (**c**) GCD curves in KOH at different current densities; and (**d**) the corresponding specific capacitances.

The supercapacitor performance of the Rc@AuNPs hybrid material was investigated in acidic, neutral, and basic electrolytes using the GCD technique. Measurements were performed in the potential range of −0.3 V to +0.5 V in KOH and 0.0 V to +0.8 V in Na_2_SO_4_ and H_2_SO_4_ at a constant current density of 0.6 A/g. Specific capacitance (C_sp_) values were calculated from the corresponding GCD curves (Figure 8).

GCD analyses revealed distinct capacitive behaviors depending on the electrolyte type (Figure 8a). The C_sp_ values were 348.34 F/g in KOH, 133.23 F/g in Na_2_SO_4_, and 107.69 F/g in H_2_SO_4_, demonstrating that Rc@AuNPs achieved the highest energy storage capacity and superior electrochemical performance in a basic environment. This enhanced performance in KOH can be attributed to stronger interactions between AuNPs and plant-extract-derived functional groups, improved ion transport, and reduced internal resistance (as confirmed later by EIS ). The slight curvature in the KOH GCD curves indicates pseudocapacitive contributions arising not only from double-layer capacitance but also from rapid surface-controlled redox reactions between AuNPs and functional groups [33,37]. These faradic contributions occur simultaneously with non-faradaic processes, enhancing total capacitance and the synergistic energy-storage effect of the hybrid electrode.

The effect of current density on capacitive behavior was further evaluated in KOH at 0.2–0.6 A/g (Figure 8c,d). Specific capacitance decreased with increasing current density, from 348.34 F/g at 0.2 A/g to 120.57 F/g at 0.6 A/g. This decrease results from insufficient time for electrolyte ions to diffuse throughout the electrode’s porous structure at higher currents, preventing full utilization of active sites. At lower current densities, ions have sufficient time to access all active sites, enabling effective charge storage and higher *C_sp_* values.

**Figure 9:**
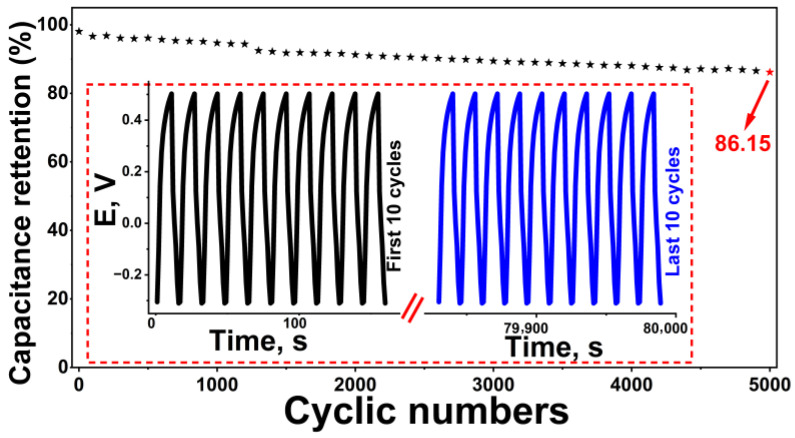
Coulombic efficiency retention of the symmetric Rc@AuNPs-based supercapacitor in KOH electrolyte over extended cycling (presented as * line). GCD curves for the first 10 and last 10 cycles of 5000 charge–discharge cycles at a current density of 0.6 A g_−1_ in aqueous KOH (shown in the dashed box).

The practical applicability of energy storage devices depends largely on long cycle life and electrochemical stability. In this context, the cyclic performance of the supercapacitor designed using the Rc@AuNPs hybrid material was investigated for 5000 charge–discharge cycles at a current density of 0.6 A g^−1^ in an aqueous KOH electrolyte (Figure 9). The results revealed that the electrode material exhibited exceptional stability, retaining 86.15% of its initial *C_sp_* value. This value is significantly higher than the capacity retention rates reported for AuNPs-based carbon hybrids in the literature and confirms the long-term structural integrity of the hybrid system [34,38,39].

The high morphological consistency between the GCD curves for the first 10 and last 10 cycles, presented in Figure 9, is consistent with low polarization and minimal capacity fading in the symmetric two-electrode configuration. This behavior is attributed to the synergistic effect of the electrochemical contributions from the redox-active functional groups of the *R. coriaria* plant extract and the high conductivity and mechanical stability provided by AuNPs. Similarly, the stability-enhancing role of AuNPs in conductive polymers or carbon frameworks has been previously reported [37,40,41]. These results demonstrate that the robust network structure formed by AuNPs effectively absorbs mechanical stresses during cycling, preventing structural deformation and maintaining continuous ion diffusion pathways. Furthermore, the use of *R. coriaria* plant extract as a reducing and capping agent in the green synthesis process provides an environmentally sustainable production approach. In recent years, the applicability of similar green synthesis protocols in energy storage systems has increased, and the advantages of plant-extract-based metal nanoparticles, such as high electrochemical stability and environmentally friendly production, have been highlighted [36,37,42].

**Figure 10:**
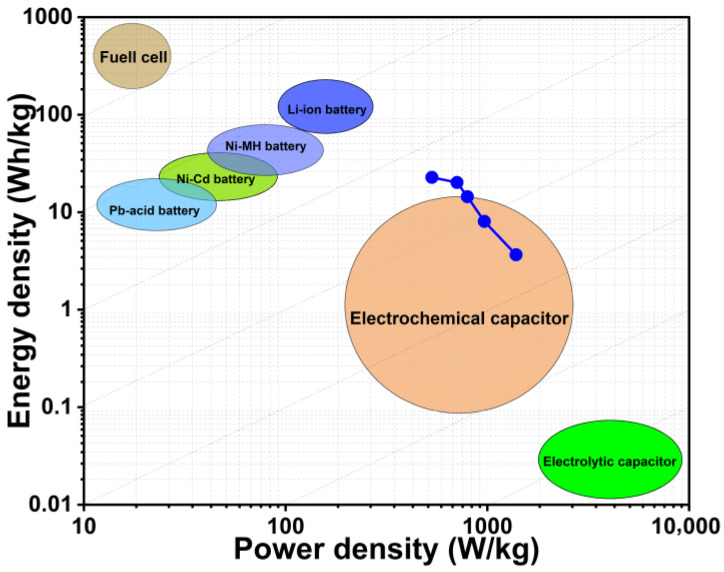
Ragone plot showing the energy density versus power density of the symmetric Rc@AuNPs-based supercapacitor.

The Ragone diagram, commonly used to illustrate the performance limits of energy storage systems, highlights the fundamental trade-off between a device’s energy density and power density [25,43]. In this study, the performance of a symmetric two-electrode supercapacitor based on Rc@AuNPs hybrid material, measured in KOH electrolyte, was evaluated using Ragone curves (Figure 10).

It was observed that the calculated power density values increased from 530.77 Wh/kg to 1388 Wh/kg, while the energy density decreased from 22.78 Wh/kg to 3.67 Wh/kg. This inverse relationship is characteristic of supercapacitors, enabling rapid energy delivery at high power demands while partially reducing the stored energy. The highest measured energy density (22.78 Wh/kg) exceeds the typical range of conventional electrochemical capacitors and approaches the lower limits of lithium-ion batteries [37,43]. Simultaneously, power densities of up to 1388 Wh/kg are consistent with the material’s high conductivity and rapid ion adsorption/desorption kinetics. This balanced performance is enabled by the synergistic combination of redox-active functional groups from the *R. coriaria* plant extract and efficient charge transfer with low internal resistance provided by the AuNPs [34,44,45].

Consequently, the Rc@AuNPs-based supercapacitor exhibits a “bridge material” property, combining the advantages of both systems by offering higher energy density than conventional capacitors and higher power density than batteries. This well-balanced performance profile in the symmetric two-electrode configuration is particularly promising for hybrid energy systems and electric vehicle applications, where high power output and mesoscale energy storage are critical.

### 3.4. Electrochemical Impedance Spectroscopy Analysis

**Figure 11:**
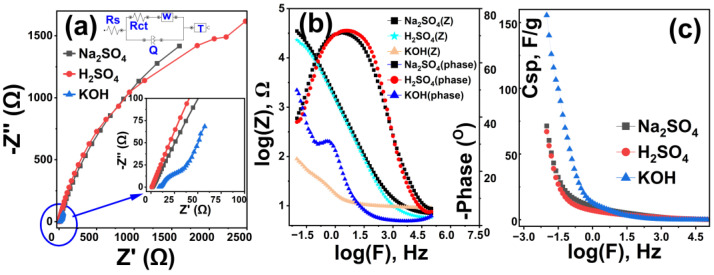
Electrochemical impedance analysis of the Rc@AuNPs-based supercapacitor in different electrolytes: (**a**) Nyquist plots, (**b**) Bode phase diagrams, and (**c**) specific capacitance values derived from EIS measurements.

To evaluate the electrochemical behavior of the Rc@AuNPs hybrid material and the effect of electrolyte selection on its performance, EIS measurements were performed in three different electrolyte environments (KOH: blue, H_2_SO_4_: red, and Na_2_SO_4_: black) (Figure 11). All measurements were obtained at open-circuit potential, with a signal amplitude of 10 mV and a frequency sweep from 0.01 Hz to 100 kHz [46]. Fitting of the EIS data was performed using the [Rs[Rct W]Q)T] equivalent circuit model.

The findings clearly reveal that electrolyte chemistry plays a critical role in determining the ESR, capacitive behavior, and energy-storage capacity of the Rc@AuNPs hybrid system [11,34,47]. ESR values calculated from Nyquist diagrams (Figure 11a) showed that the lowest internal resistance was obtained in KOH medium, at 24.43 Ω. This finding is consistent with the high ionic conductivity of the KOH solution, indicating efficient charge transfer at the electrode/electrolyte interface. In contrast, the high ESR values observed in Na_2_SO_4_ (64.42 Ω) and H_2_SO_4_ (67.96 Ω) media indicate that ion transport in the system is limited and charge-transfer kinetics are slowed. To further evaluate the influence of electrolyte type, the effect on capacitive properties was investigated using Bode phase diagrams (Figure 11b). The phase angle closest to the ideal capacitor behavior (90°) was obtained in the KOH electrolyte at 52.42°, indicating a strong capacitive nature of the system [11,37,48]. In contrast, the low phase angles measured in Na_2_SO_4_ (39.65°) and H_2_SO_4_ (38.05°) environments suggest that capacitive behavior is partially impaired in these systems. Furthermore, resistance values derived from Bode diagrams (KOH: 1.95 Ω; Na_2_SO_4_: 4.36 Ω; H_2_SO_4_: 4.59 Ω) were found to be consistent with the ESR results and further supported the superior conductivity properties of the KOH electrolyte.

To evaluate the energy-storage capacity, *C_sp_* values were calculated (Figure 11c) [38,46]. The results show that the choice of electrolyte has a significant impact on energy-storage performance. At the lowest frequency, the highest *C_sp_* value of 155.56 F/g was obtained in KOH medium, while this value was approximately halved in H_2_SO_4_ (71.62 F/g) and Na_2_SO_4_ (67.14 F/g) electrolytes. These differences are thought to be due to the hydration properties of the electrolyte ions, their ion sizes, and their interaction with the pore morphology of the hybrid electrode [11,38,47,49].

Overall, EIS analyses clearly demonstrated that the KOH electrolyte is the most suitable operating environment for the Rc@AuNPs hybrid material. The low ESR, high phase angle, and superior specific capacitance values obtained in the KOH environment confirm that this system offers high efficiency for supercapacitor applications. Furthermore, the gold nanoparticles incorporated into the hybrid structure are believed to significantly enhance the observed performance by improving the electrode’s overall conductivity [50,51,52]. In addition, the high *C_sp_* value obtained by the EIS technique in KOH medium is in full compliance with the electrochemical performance results determined by the CV and GCD techniques, which collectively confirms the superior capacitive behavior of the Rc@AuNPs electrode.

## 4. Conclusions

In this study, gold nanoparticles (Rc@AuNPs) were successfully synthesized using bioactive components derived from the *Rhus coriaria* (sumac) plant via an environmentally friendly, sustainable, and low-cost method. The polyphenolic compounds and phytochemical reducing agents in *R. coriaria* extract played an active role in the reduction of Au^3+^ ions and surface stabilization of the nanoparticles, achieving a green synthesis approach without using any toxic chemical reducers.

Structural and electrochemical characterizations demonstrated that Rc@AuNP nanoparticles are a promising electrode material with high energy storage performance. The electrochemical behavior of Rc@AuNPs in different electrolytes was evaluated comprehensively using cyclic voltammetry (CV), galvanostatic charge–discharge (GCD), and electrochemical impedance spectroscopy (EIS). The specific capacitance (C_sp_) values were 129.48 F/g, 156.32 F/g, and 280.37 F/g in H_2_SO_4_, Na_2_SO_4_, and KOH, respectively, while GCD measurements yielded C_sp_ values of 107.69 F/g, 133.23 F/g, and 348.34 F/g in acidic (H_2_SO_4_), neutral (Na_2_SO_4_), and basic (KOH) environments, respectively. These results indicate that Rc@AuNPs achieve highest capacitive performance in basic media, with KOH providing the most suitable electrolyte due to its high ionic conductivity.

ESR values obtained by EIS were 24.43 Ω, 67.96 Ω, and 64.42 Ω for KOH, H_2_SO_4_, and Na_2_SO_4_, respectively. The low ESR in KOH indicates efficient charge transfer at the electrode/electrolyte interface and low internal resistance, which correlates with the high capacitance observed.

Overall, the high specific capacitance, low internal resistance, and efficient charge transfer of Rc@AuNPs in KOH demonstrate that this material is a promising candidate for high-performance supercapacitors and energy storage systems. Furthermore, the environmental sustainability, green chemistry compatibility, and high electrochemical stability of biosynthesized Rc@AuNPs suggest that they could play a significant role in future renewable energy technologies and environmentally friendly energy storage applications.

## Data Availability

The original contributions presented in this study are included in the article. Further inquiries can be directed to the corresponding authors.
